# Domains of common mental disorders in women reporting intimate partner violence*

**DOI:** 10.1590/1518-8345.2740.3099

**Published:** 2018-11-29

**Authors:** Ariane Gomes dos Santos, Claudete Ferreira de Souza Monteiro

**Affiliations:** 1 Universidade Federal do Piauí, Departamento de Enfermagem, Teresina, PI, Brasil.; 2 Instituto Federal de Educação, Ciência e Tecnologia do Piauí, Departamento de Saúde, Teresina, PI, Brasil.

**Keywords:** Women, Mental Disorders, Intimate Partner Violence, Mental Health, Nursing, Public Health

## Abstract

**Objective:**

to verify associations between the types of intimate partner violence and the domains of common mental disorders in women.

**Method:**

cross-sectional study with 369 women. The information was obtained through the instruments Self-Reporting Questionnaire and Conflict Tactic Scales. To analyze the data, Pearson’s Chi-Square test, Fisher’s exact test and Odds Ratio were used.

**Results:**

women who reported physical abuse with and without sequela were respectively 2.58 and 3.7 times more likely to have symptoms of anxious depressed mood. The chances of experiencing symptoms of decreased vital energy increased by 2.27 times with psychological aggression, 3.06 times with physical abuse without sequelae and 3.13 times with physical abuse with sequelae. Somatic symptoms did not show statistical association with the types of violence. The propensity to develop symptoms of depressive thoughts increased 3.11 times with psychological aggression, 6.13 times with physical aggression without sequelae, 2.47 times with sexual coercion and 7.3 times with physical aggression with sequelae.

**Conclusion:**

the types of intimate partner violence are strongly associated with the domains of common mental disorders in women. This finding may contribute to more accurate interventions by health professionals to women victims of violence.

## Introduction

Common mental disorders are manifested by symptoms of depression, anxiety and somatizations that interfere with the quality of life of the individuals presenting them, although they do not meet the criteria necessary for the diagnosis of mood disorders recommended by the Diagnostic and Statistical Manual of Mental Disorders, 5th edition (DSM-V) nor by the 10th revision of the International Statistical Classification of Diseases and Related Health Problems (ICD-10)^(^ ^1^ ^-^ ^3^ ^)^. These disorders are highly prevalent and affect people from all over the world^(^ ^4^ ^-^ ^6^ ^)^. According to studies, this prevalence ranges from 15.0% to 50.3%^(^ ^7^ ^-^ ^10^ ^)^.

In order to better explore common mental disorders, we used the Self-Reporting Questionnaire (SRQ-20), which subdivides the symptoms of these disorders into four domains: anxious depressive mood, somatic symptoms, decreased vital energy and depressive thoughts^(^ ^11^ ^)^.

Anxious depressive mood is characterized by symptoms such as nervousness, tension, worry, sadness, crying and being easily scared. People with somatic symptoms may experience headaches, insomnia, stomach discomfort, poor digestion, poor appetite, and shaking of the hands. Decreased vital energy corresponds to symptoms such as being easily fatigued, difficulties in making decisions or in having satisfaction in one’s tasks, difficulty in thinking and suffering with work activities. People who feel unable to play a useful role in life, lose interest in things, feel useless and think of ending their own lives fall within the domain of depressive thoughts^(^ ^11^ ^)^.

Mental health risks must be seen from a gender perspective, which influences the expression of suffering between men and women^(^ ^12^ ^)^. Common mental disorders are more frequently present among women^(^ ^1^ ^,^ ^13^ ^)^. Another issue that relates to common mental disorders in women is violence^(^ ^14^ ^-^ ^15^ ^)^.

Thus, the present research is justified by the importance of identifying the symptoms of domains of common mental disorders in women who report different types of intimate partner violence, since the early discovery of these disorders is essential to minimize damage to physical and mental health. With trained and sensitive professionals to identify symptoms of these domains among their clientele, it will be possible to seek ways to address this problem and empower women beaten by their partners through an effective approach^(^ ^16^ ^)^.

In view of this problem, the following research question was raised: Are the different types of intimate partner violence associated with the domains of common mental disorders in women? This study aimed to verify associations between the types of intimate partner violence and the domains of common mental disorders in women.

## Method

This is a cross-sectional study carried out in five cities of Piauí (Teresina, Parnaíba, Picos, Floriano and Bom Jesus), selected for being headquarters in the health macro-regions of the state, set in the Regionalization Master Plan (RMP).

The data were obtained from the database of the study: “Violence, alcohol consumption and drugs in the female universe: prevalence, risk factors and mental health consequences”, funded by the National Council for Scientific and Technological Development (CNPq). All the women who composed the database were used for this study.

The sample met the following inclusion criteria: women aged 20 to 59 years who were attended in nursing consultations in the Basic Health Units of the mentioned cities. The adopted exclusion criterion was presenting auditory or verbal difficulty detected at the time of the invitation according to the researcher’s observation, considering the importance of these functions for the application of the instruments.

Data from the last census conducted by the Brazilian Institute of Geography and Statistics (IBGE) in 2010 showed that in the cities where this study was conducted the population of women aged 20 to 59 years was 347,414. Thus, for the calculation of the minimum sample required, the formula for infinite populations was used based on the population proportion: n= (Z_α/2_)^2^. p.q / E^2^, where *Z*_α*/2*_ is the point of the normal curve that corresponds to the desired confidence interval (95%); *p* comprises the proportion of individuals belonging to the category to be studied, for which the estimated prevalence of non-psychotic mental disorders was considered 39.4%^(^ ^17^ ^)^; *q* is the proportion not belonging to the category (q=1– p); and *E* consists of the maximum error of estimate (5%).

The minimum sample found by means of the calculation was 367; however, 369 women were used for this investigation. Thus, this sample was sufficient to allow internal validity of the study, since it enabled generating results that reflect what occurs in the target population.

This sample was selected randomly and proportionally stratified according to the number of attendances of the basic health units of each studied city. The number of basic units and women in each city was drawn in Excel 2010 software in order to avoid selection bias.

Data collection took place from August 2015 to May 2016 by previously trained staff. For this work, the data were collected by the instruments: Conflict Tactic Scales (CTS-2), which verifies the self-report of intimate partner violence; and the Self-Reporting Questionnaire (SRQ-20), which investigates the suspicion of common mental disorders. Both of them are in the public domain, translated into Portuguese language, adapted to Brazilian culture and validated in Brazil.

Regarding the indicators of the psychometric strength of the instruments, SRQ-20 has a good performance in evaluating the suspicion of common mental disorders, showing that, despite the multiple nature of the emotional disorders, the instrument is capable of identifying factors with easy applicability and reliability, indispensable for the tracking of the mental health of the interviewees^(^ ^18^ ^)^. The CTS-2 scales have good fidelity, validity, easy comprehension and application indices to verify the self-report of intimate partner violence, being used in diverse spatial and social realities, constituting as a reliable and effective instrument^(^ ^19^ ^)^.

The CTS-2 are subdivided into five dimensions, with two subscales each: negotiation (cognitive and emotional); psychological aggression (severe and minor); physical abuse without sequelae (severe and minor); physical abuse with sequelae (severe and minor); and sexual coercion (severe and minor)^(^ ^20^ ^-^ ^21^ ^)^. The positive response to at least one item from each subscale was considered as presence of violence. The negotiation subscale was not analyzed in this study.

The SRQ-20 is composed of 20 questions measured in a dichotomized nominal scale: (1) yes or (0) no. The items of this scale are divided into four domains: anxious depressive mood (4 items); somatic symptoms (6 items); decreased vital energy (6 items); depressive thoughts (4 items)^(^ ^11^ ^)^. The positive response to at least one item from each domain was considered as presence of symptoms of a particular domain.

Thus, the predictive variables of this study were the types of intimate partner violence (psychological aggression, physical abuse without sequelae, physical abuse with sequelae and sexual coercion) in the minor and severe degrees, whereas the outcomes were the domains of common mental disorders (anxious depressive mood, somatic symptoms, decreased vital energy and depressive thoughts). All variables were analyzed at the qualitative level of measurement.

Data analysis for this work was carried out from October to December 2017. We used the statistical software R version 3.4.1 in the exploration and statistical analysis of the data, and bivariate analysis to verify the existence of associations through the chi-square test or Fisher’s exact test, when all the presuppositions of the chi-square test were not satisfied. We used a 5% level of significance. To quantify the intensity of the association, the odds ratio and respective intervals were used with 95% confidence level.

Considering the ethical aspects, the analysis of information began after the approval of the Ethics Research Committee (opinion no. 2,379,740/2017) and all the participants signed the Informed Consent Form in two copies.

## Results

**Figure 1:**
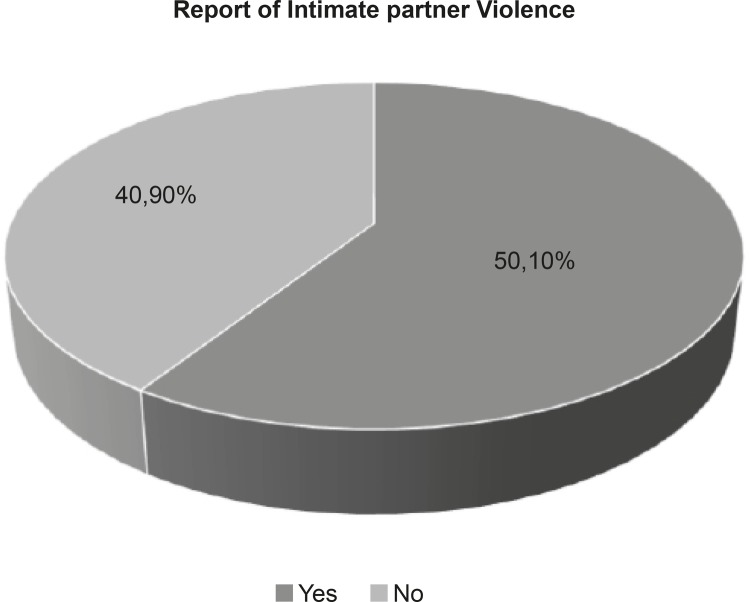
Prevalence of women who reported experiencing intimate partner violence, Teresina, PI, Brazil, 2015-2016

Figure 1 showed that the prevalence of intimate partner violence among women interviewed was 59.10%.

Table 1 showed that women who reported having suffered physical abuse without sequelae in a miner and severe degree presented, respectively, 1.82 and 2.58 times more chances of having symptoms of anxious depressive mood, when compared with those who did not report it. Women who reported having suffered physical abuse with minor sequelae were 3.7 times more likely to have symptoms of depressed mood than those who did not report it.

**Table 1:** Association between self-report of different types of intimate partner violence, in minor and severe degrees, and symptoms of depressive mood, Teresina, PI, Brazil, 2015-2016

Reported violence	Symptoms of Depressive Anxious Mood				Total (369)	Odds (95%CI)*

	Yes (293)		No (76)			
	
	n	%	n	%		
Psychological Aggression (Minor)						
Yes	183	81.7	41	18.3	224	1.42 (0.85; 2.36)
No	110	75.9	35	24.1	145	
Psychological Aggression (Severe)						
Yes	125	81.7	28	18.3	153	1.28 (0.76; 2.15)
No	168	77.8	48	22.2	216	
Physical abuse without sequelae (Minor)						
Yes	101	85.6	17	14.4	118	1.82 (1.01; 3.30)
No	192	76.5	59	23.5	251	
Physical abuse without sequelae (Severe)						
Yes	53	89.8	6	10.2	59	2.58 (1.06; 6.25)
No	240	77.4	70	22.6	310	
Sexual coercion (Minor)						
Yes	42	84.0	8	16.0	50	1.42 (0.64; 3.17)
No	251	78.7	68	21.3	319	
Sexual coercion (Severe)						
Yes	22	95.7	1	4.3	23	6.1 (0.81; 45.45)
No	271	78.3	75	21.7	346	
Physical abuse with sequelae (Minor)						
Yes	50	92.6	4	7.4	54	3.7 (1.29; 10.63)
No	243	77.1	72	22.9	315	
Physical abuse with sequelae (Severe)						
Yes	23	88.5	3	11.5	26	2.07 (0.61; 7.09)
No	270	78.7	73	21.3	343	

*(95%CI) = 95% of Confidence Interval

There was no statistically significant association between the types of violence and the presence of somatic symptoms among the women interviewed (Table 2).

**Table 2:** Association between self-report of different types of intimate partner violence, in minor and severe degrees, and somatic symptoms, Teresina, PI, Brazil, 2015-2016

Reported violence	Somatic Symptoms				Total (369)	Odds (95%CI)*

	Yes (291)		No (78)			
	
	n	%	n	%		
Psychological Aggression (Minor)						
Yes	183	81.7	41	18.3	224	1.53 (092; 2.53)
No	108	74.5	37	25.5	145	
Psychological Aggression (Severe)						
Yes	29	19.0	124	81.0	153	1.25 (0.76; 2.10)
No	49	22.7	167	77.3	216	
Physical abuse without sequelae (Minor)						
Yes	98	83.1	20	16.9	118	1.47 (0.84; 2.58)
No	193	76.9	58	23.1	251	
Physical abuse without sequelae (Severe)						
Yes	52	88.1	7	11.9	59	2.21 (0.96; 5.08)
No	239	77.1	71	22.9	310	
Sexual coercion (Minor)						
Yes	43	86.0	7	14.0	50	1.76 (0.76; 4.08)
No	248	77.7	71	22.3	319	
Sexual coercion (Severe)						
Yes	21	91.3	2	8.7	23	2.96 (0.68; 12.82)
No	270	78.0	76	22.0	346	
Physical abuse with sequelae (Minor)						
Yes	45	83.3	9	16.7	54	1.40 (0.65; 3.01)
No	246	78.1	69	21.9	315	
Physical abuse with sequelae (Severe)						
Yes	22	84.6	4	15.4	26	1.51 (0.51; 4.52)
No	269	78.4	74	21.6	343	

*(95%CI) = 95% of Confidence Interval

Table 3 shows that women who reported having suffered psychological aggression, to a minor degree, were 2.07 times more likely to have symptoms of decreased vital energy than those who did not report it. This propensity increased to 2.27 times among women who reported having experienced severe psychological aggression.

**Table 3:** Association between self-report of types of intimate partner violence, in minor and severe degrees, and symptoms of decreased vital energy, Teresina, PI, Brazil, 2015-2016

Reported violence	Symptoms of Decreased Vital Energy				Total (369)	Odds (95%CI)*

	Yes (250)		No (119)			
	
	n	%	n	%		
Psychological Aggression (Minor)						
Yes	166	74.1	58	25.9	224	2.07 (1.33; 3.25)
No	84	57.9	61	42.1	145	
Psychological Aggression (Severe)						
Yes	119	77.8	34	22.2	153	2.27 (1.42; 3.62)
No	131	60.6	85	39.4	216	
Physical abuse without sequelae (Minor)						
Yes	93	78.8	25	21.2	118	2.23 (1.33; 3.70)
No	157	62.5	94	37.5	251	
Physical abuse without sequelae (Severe)						
Yes	50	84.7	9	15.3	59	3.06 (1.45; 6.45)
No	200	64.5	110	35.5	310	
Sexual coercion (Minor)						
Yes	37	74.0	13	26.0	50	1.42 (0.72; 2.78)
No	213	66.8	106	33.2	319	
Sexual coercion (Severe)						
Yes	18	78.3	5	21.7	23	1.77 (0.64; 4.88)
No	232	67.1	114	32.9	346	
Physical abuse with sequelae (Minor)						
Yes	46	85.2	8	14.8	54	3.13 (1.43; 6.85)
No	204	64.8	111	35.2	315	
Physical abuse with sequelae (Severe)						
Yes	22	84.6	4	15.4	26	2.77 (0.93; 8.26)
No	228	66.5	115	33.5	343	

*(95%CI) = 95% of Confidence Interval

Participants who reported having suffered physical abuse without sequelae, to a minor degree, were 2.23 times more likely to present symptoms of decreased vital energy when compared to those who did not report it. Women who reported having experienced this same type of violence in a severe degree were 3.06 times more likely to present symptoms of decreased vital energy. Those that reported physical abuse with sequelae, to a minor degree, were 3.13 times more likely to have symptoms of decreased vital energy.

Table 4 shows that women who reported psychological aggression, to a minor degree, are 2.93 more likely to have depressive thoughts than those who do not report it. Women who reported severe psychological violence are 3.11 times more likely to have depressive thoughts in relation to those who did not report it.

**Table 4:** Association between self-report of different types of intimate partner violence, in minor and severe degrees, and symptoms of depressive thoughts, Teresina, PI, Brazil, 2015-2016

Reported violence	Symptoms of Depressive Thoughts				Total 369	Odds (95%CI)*

	Yes (291)		No (78)			
	
	n	%	n	%		
Psychological Aggression (Minor)						
Yes	77	34.4	147	65.6	224	2.93 (1.72; 4.98)
No	22	15.2	123	84.8	145	
Psychological Aggression (Severe)						
Yes	61	39.9	92	60.1	153	3.11 (1.93; 5.00)
No	38	17.6	178	82.4	216	
Physical abuse without sequelae (Minor)						
Yes	54	45.8	64	54.2	118	3.86 (2.38; 6.29)
No	45	17.9	206	82.1	251	
Physical abuse without sequelae (Severe)						
Yes	36	61.0	23	39.0	59	6.13 (3.51; 11.11)
No	63	20.3	247	79.7	310	
Sexual coercion (Minor)						
Yes	22	44.0	28	56.0	50	2.47 (1.34; 4.57)
No	77	24.1	242	75.9	319	
Sexual coercion (Severe)						
Yes	10	43.5	13	56.5	23	2.22 (0.94; 5.24)
No	89	25.7	257	74.3	346	
Physical abuse with sequelae (Minor)						
Yes	33	61.1	21	38.9	54	5.92 (3.22; 10.87)
No	66	21.0	249	79.0	315	
Physical abuse with sequelae (Severe)						
Yes	18	69.2	8	30.8	26	7.30 (3.05; 17.24)
No	81	23.6	262	76.4	343	

*(95%CI) = 95% of Confidence Interval

Women who reported having suffered minor degree of physical aggression without sequelae were 3.86 times more likely to develop depressive thoughts than those who did not report it. This propensity increased to 6.13 times when the degree became severe. Those who reported minor sexual coercion were 2.47 times more likely to present depressive thoughts when compared to those who did not report it.

Women who reported physical aggression with sequelae, to a minor degree, were 5.92 times more likely to develop depressive thoughts compared to those who did not report it. When the degree of this violence became severe, the chances of the woman presenting symptoms of depressive thoughts increased 7.3 times.

## Discussion

Psychological, physical, and sexual violence at minor and severe intensities related in different ways to symptoms of the four domains of common mental disorders.

This study revealed that women who reported suffering physical abuse without severe sequelae and physical abuse with sequelae to a minor degree were more likely to present symptoms of anxious depressive mood. Anxious depressive mood is characterized by symptoms such as nervousness, tension, worry, sadness, crying and being easily scared^(^ ^11^ ^)^.

Sadness may be considered in some individuals as an early stage of the depressive picture, which allows to consider this as an “at risk” mental state^(^ ^22^ ^)^. Thus, the identification of sadness in the general population can be useful to detect subjects vulnerable to the development of mental disorders. This could help to propose goals and strategies for the early prevention of this condition^(^ ^23^ ^)^. For the victim of intimate partner violence, aggression by the person with whom they are emotionally involved may generate feelings of impotence, decreased self-esteem and depression^(^ ^24^ ^)^.

Depressive disorders are greater in women experiencing intimate partner violence compared to non-victims. This same author describes in his study that women who suffer intimate partner violence are almost twice as likely to have problems related to mental health^(^ ^25^ ^)^.

Physical and verbal abuse can generate intense self-recrimination. However, despite the suffering generated by the aggressor, women fail to see their condition as victims; instead, they tend to feed a sense of guilt for the violence suffered. As a result, these women find it difficult to love, have fun, study and look after their children^(^ ^26^ ^)^.

A study of 298 women who had been victims of domestic violence by a male intimate partner showed that having been threatened with a firearm, regardless of other forms of intimate partner violence, is related to symptoms of post-traumatic stress disorder in women. Approximately one quarter of the sample (24.2%) had experienced a threat with a firearm throughout their relationship and 12.5% feared their partners would use a firearm against them^(^ ^27^ ^)^. This fear surrounding the lives of intimate partner victims may be a preponderant factor for the emergence of common mental disorders.

A study in Sweden also showed similar results by pointing that that women who were exposed to physical and sexual violence were 3.78 times more likely to present depressive symptoms than women who had not been exposed to such violence^(^ ^28^ ^)^.

A survey of 775 pregnant women in São Paulo showed that domestic violence and mental disorders were highly correlated. About 27.15% of the women interviewed had experienced domestic violence and about 38.24% of them were diagnosed with mental disorders. The main association found was between anxiety and physical violence^(^ ^29^ ^)^. Other research has similar results showing that 31.6% of women who had been assaulted by their partners reported experiencing at least two symptoms of depression^(^ ^28^ ^)^.

It has been observed that the symptoms of anxious depressive mood are related to physical abuse with and without sequelae. This does not imply that other types of violence should not be considered, since all of them may, in some way, have an impact on the lives of victims.

This research did not present significant results regarding the association between types and intensities of intimate partner violence and somatic symptoms. This domain is characterized by symptoms such as headaches, insomnia, stomach discomfort, poor digestion, lack of appetite and shaking in the hands^(^ ^11^ ^)^.

Intimate partner violence is a common occurrence and is particularly prevalent among women. It is related to mental health problems including depression, anxiety, post-traumatic stress disorder, substance use, eating disorders and a number of psychosomatic conditions^(^ ^30^ ^)^.

The same disorder can manifest itself differently in different people^(^ ^31^ ^)^. The literature shows that intimate partner violence is strongly associated with sleep disorders and mental health^(^ ^32^ ^)^. Individuals with mental disorders also present the aggravation of having impaired social relationships^(^ ^33^ ^)^. Thus, common psychiatric disorders in victims of intimate partner violence may include somatic symptoms, anxiety, insomnia, social dysfunction and depression^(^ ^34^ ^)^.

Even though this study did not show statistically significant associations between any type of intimate partner violence and somatic symptoms, it is considered relevant to cite this domain, since many women and health professionals take into account only the physical aspects of pathologies, without considering that these effects may be somatic symptoms of psychological disorders.

There was significant associations between psychological aggression, in minor and severe degrees; physical abuse without sequelae, in minor and severe degrees; and physical abuse with sequelae, in minor degree, with the presence of symptoms of decreased vital energy. Also, when the psychological and physical violence were severe, the woman’s chances of experiencing symptoms of decreased vital energy increased.

This domain is characterized by symptoms such as being easily fatigued, difficulties in making decisions or in having satisfaction in their tasks, difficulty in thinking and suffering with work activities^(^ ^11^ ^)^.

A study conducted in Sweden with 573 women who experienced intimate partner violence pointed out that, among the women interviewed, 45.7% reported experiencing visible fatigue and tiredness every day or once a week, while 29.7% reported difficulties in falling asleep and 18.3% had difficulty concentrating in the last 12 months^(^ ^28^ ^)^.

Physical violence can have as a consequence symptoms of decreased vital energy through difficulty awakening, and less interest in feeding and socializing with others^(^ ^35^ ^)^. A study with more than 24,000 women found an association between intimate partner violence and poor health, difficulty walking and performing daily activities, memory loss, dizziness and reproductive problems^(^ ^36^ ^)^.

Another study with 2,091 women showed that when it comes to the pattern of intimate partner violence, psychological aggression is more frequent than physical aggression, sexual coercion or injury^(^ ^34^ ^)^.

The severity of the aggressions contributes to increase the chances of the woman presenting symptoms of decreased vital energy. Thus, a careful investigation by the health team in cases of intimate partner violence is of utmost importance in order to seek early intervention in such cases, so as to avoid aggravating the consequences resulting from violent acts.

Depressive thoughts were the domain of common mental disorders that showed a significant association with more types and intensities of intimate partner violence, among them psychological aggression, in minor and severe degrees; physical aggression without sequelae, in minor and severe degrees; physical aggression with sequelae, in minor and severe degrees; and sexual coercion in minor degree. The severity of the aggressions that had statistically significant association increased the chances of women presenting symptoms of depressive thoughts.

People who feel unable to play a useful role in life, lose interest in things, feel useless and think of ending their lives fall within this domain^(^ ^11^ ^)^.

In a study developed in France with 38,694 individuals, sadness was associated with psychiatric problems and suicide^(^ ^22^ ^)^. The risk of suicide is significantly higher among individuals with poor physical and/or mental health^(^ ^33^ ^)^. Another study showed that 42% of women who had experienced intimate partner violence reported suicidal ideation and 31% reported having attempted suicide at some point in their life^(^ ^37^ ^)^. A recent systematic review also pointed out that women who were exposed to intimate partner violence may be at high risk of death from suicide^(^ ^38^ ^)^.

A study of 134 women in a city on the Gulf Coast-USA showed that 28% of the sample reported a suicide attempt and 20% reported suicidal ideation. Correlations indicated that suicidal ideation and suicide attempts were largely associated with coercive control by the partner^(^ ^39^ ^)^.

Psychological abuse, including the control of one’s behavior, is as harmful to a woman’s health as other forms of intimate partner violence. It is used by the perpetrator to gain obedience and dependence, and generates deprivation of important aspects of the women’s daily life, such as economic resources, social life and the right to have a paid work^(^ ^40^ ^)^.

A study of 390 adult women in Pernambuco showed that the incidence of common mental disorders was 44.6% among women who reported having suffered intimate partner violence in the last 12 months and 43.4% in the last seven years. Mental disorders have remained associated with psychological violence, even in the absence of physical or sexual violence. However, when psychological violence was concomitant with physical or sexual violence, the risk of common mental disorders was even greater^(^ ^41^ ^)^.

Importantly, women exposed to behavioral control are more likely to present symptoms of depression^(^ ^28^ ^)^, which is classified by the World Health Organization as the main contributor to suicide deaths^(^ ^24^ ^)^. A longitudinal, nationally representative study developed in Korea showed that women victims of intimate partner violence are four times more likely to have symptoms of depression and approximately seven times more likely to have suicidal ideation when compared to women who did not experience partner violence intimate^(^ ^42^ ^)^.

Women exposed to physical and sexual conditions of violence are also more susceptible to depressive symptoms^(^ ^28^ ^)^. A study of 1,049 women in Tanzania corroborates this information by showing that physical and sexual violence were associated with increased report of symptoms of poor mental health^(^ ^43^ ^)^.

However, since couples often do not mention issues such as sexual violence, even when they are present^(^ ^26^ ^)^, this contributes to the fact that women who have experienced sexual abuse are also at higher risk of suicide^(^ ^44^ ^)^.

A study has shown that sexual violence was associated with the greater severity of the symptoms of post-traumatic stress disorder^(^ ^28^ ^)^. In another study conducted in Australia with 230 adult women, more than half of the sample had experienced at least one incident of sexual violence. Most reported unwanted caresses and being forced to sex due to pressure and coercion by the partner. Another form of sexual violence found in that research was the fact that the partner refuses to use condoms when invited to do so. Women who have experienced sexual violence were more likely to be anxious, depressed, depressed or hopeless^(^ ^45^ ^)^.

The prevalence of anxiety and depression symptoms among these women suggests that all forms of sexual violence should be considered as a potential major factor for the suspicion of common mental disorders^(^ ^45^ ^)^. Stressful life events, especially personal losses, neglect and physical, emotional or sexual abuse, increase the likelihood of mental illness by making the brain response more intense and hypersensitive to stress^(^ ^31^ ^)^.

Another study conducted in Australia with 1,163 women found that the severity of the injury caused by intimate partners is greater than that of injury caused by others, so that moderate or severe injury was observed in 30.4% of sexually abused women by intimate partners, 16.4% by strangers and 14.9% by friends/acquaintances^(^ ^46^ ^)^.

A study conducted in Rasht, Iran, with 2,091 married women pointed out that the most prevalent type of intimate partner violence was psychological aggression, but physical aggression, sexual coercion or injury were also present. The domains of common mental disorders in victims of intimate partner violence were significantly affected in the following aspects: somatic symptoms anxiety/insomnia, social dysfunction and depression. Psychological and sexual abuse were predictors of all these aspects of mental health, except for social dysfunction^(^ ^34^ ^)^.

Thus, the prevalence of different types of intimate partner violence among women is quite high. The findings that psychological, physical and sexual abuse are often experienced simultaneously and that all types of violence can result in mental health problems suggest that health professionals should view all victims of intimate partner violence as potentially suspected to have mental health dysfunction^(^ ^34^ ^)^.

The more severe the violence, the higher the risk of psychological trauma. More severe and recent forms of violence produce more severe symptoms of trauma, especially anxiety disorders. In the case of phobic anxiety, the symptoms disappear over time, regardless of the severity of the victimization^(^ ^47^ ^)^. This indicates that problem solving protects people from stressful life events^(^ ^31^ ^)^.

Research developed in Spain with 10,171 women, a national representative sample, showed that control behavior and current physical and sexual violence were also associated with the highest probability of reporting poor emotional health results when compared to previous violence^(^ ^48^ ^)^.

Psychological, physical and sexual violence increased the chances of women having symptoms of depressive thoughts. This was the only domain that showed association with these three types of violence. This is concerning when one considers that among the symptoms of this domain of common mental disorders is suicidal ideation. Due to the severity of the symptoms presented, it is essential that managers and health professionals seek strategies for finding and following up these women, thus helping them to coping with this problem through female empowerment.

After presenting the discussion, we suggest that more specific studies on suicidal ideation and other symptoms of depressive thoughts related to intimate partner violence among women are performed. From the in-depth knowledge on the subject, health professionals will be more able to develop more effective strategies on this subject.

The limitations of the present study include the fact that the SRQ-20 specifically traces suspected cases of common mental disorders. Although the SRQ-20 has reliable standards for prevalence studies, the ideal diagnosis would be consultation with a psychiatrist. However, this limitation does not diminish the importance of the results achieved in this study, since in identifying suspected cases, the multiprofessional team may refer the patients to a specialized consultation.

This study does not have generalization power for the general population, considering that it was developed only with adult women from five cities of Piauí, although the sample was randomly selected and representative of the target population, which allows internal validity to the study. Therefore, the development of multicentric studies on this subject with women from different regions is highly recommended.

Health professionals, especially those working in primary care and mental health area, are critical to proving care to these women. The health team should be prepared to recognize cases of intimate partner violence and common mental disorders in order to effectively attend these women so as not only to identify violence but also to report it, prevent sequelae, seek resources to organize therapeutic projects and, if necessary, direct the victims to the most appropriate support services to the situation of violence or mental health. This will help victims of intimate partner violence to adopt behaviors that help them achieve autonomy and protection of their well-being.

## Conclusion

The different types and intensities of intimate partner violence are associated with the domains of common mental disorders in women, so that there was association between psychological, physical and sexual violence (in minor and severe degrees) and symptoms of anxious depressed mood, decreased vital energy and depressive thoughts.

Therefore, it was extremely important to analyze in a specific way the type and intensity of violence that are related to the different domains of common mental disorders. This may contribute to more accurate interventions by health professionals to women victims of unique forms of violence. It is worth noting that both the minor and the severe degrees of intimate partner violence were associated with the development of symptoms of common mental disorders in their different domains.

This makes it possible to state that although the violence suffered is considered mild, it can trigger psychological degrading effects on the lives of assaulted women. This aspect deserves attention from health professionals who, in some cases, only observe severe physical aspects of intimate partner violence, without considering the psychological and sexual consequences. These consequences are often neglected because they are considered private of the couple’s personal life.
